# *TNNT2* Gene Polymorphisms Are Associated with Susceptibility to Idiopathic Dilated Cardiomyopathy in the Han Chinese Population

**DOI:** 10.1155/2013/201372

**Published:** 2013-03-17

**Authors:** Xiaoping Li, Huan Wang, Rong Luo, Haiyong Gu, Channa Zhang, Yu Zhang, Rutai Hui, Xiushan Wu, Wei Hua

**Affiliations:** ^1^Cardiac Arrhythmia Center, Cardiovascular Institute and Fuwai Hospital, Chinese Academy of Medical Sciences, Peking Union Medical College, Beijing 100037, China; ^2^The Center of Heart Development, Key Lab of MOE for Development Biology and Protein Chemistry, College of Life Science, Hunan Normal University, Changsha, Hunan 410081, China; ^3^Department of Cardiothoracic Surgery, Affiliated People's Hospital of Jiangsu University, Zhenjiang 212000, China; ^4^Sino-German Laboratory for Molecular Medicine, Key Laboratory for Clinical Cardiovascular Genetics, Ministry of Education, Cardiovascular Institute and Fuwai Hospital, Chinese Academy of Medical Sciences, Peking Union Medical College, Beijing 100037, China

## Abstract

*Background*. Idiopathic dilated cardiomyopathy (DCM) is characterized by ventricular chamber enlargement and systolic dysfunction. The pathogenesis of DCM remains uncertain, and the *TNNT2* gene is potentially associated with DCM. To assess the role of *TNNT2* in DCM, we examined 10 tagging single nucleotide polymorphisms (SNPs) in the patients. *Methods*. A total of 97 DCM patients and 189 control subjects were included in the study, and all SNPs were genotyped by matrix-assisted laser desorption/ionization time-of-flight mass spectrometry. *Results*. In the *TNNT2* gene, there was a significant association between DCM and genotype for the tagging SNPs rs3729547 (*χ* ^2^ = 6.63, *P* = 0.036, OR = 0.650, and 95% CI = 0.453–0.934) and rs3729843 (*χ* ^2^ = 9.787, *P* = 0.008, OR = 1.912, and 95% CI = 1.265–2.890) in the Chinese Han population. Linkage disequilibrium (LD) analysis showed that the SNPs rs7521796, rs2275862, rs3729547, rs10800775, and rs1892028, which are approximately 6 kb apart, were in high LD (*D*′ > 0.80) in the DCM patients. *Conclusion*. These results suggest that the *TNNT2* polymorphisms might play an important role in susceptibility to DCM in the Chinese Han population.

## 1. Introduction

Idiopathic dilated cardiomyopathy (DCM) is a cardiac muscle disease of unknown origin that is characterized by ventricular chamber enlargement and systolic dysfunction with thinning of the left ventricular wall. DCM leads to progressive heart failure and a decline in left ventricular contractile function, conduction system abnormalities, thromboembolism, and sudden or heart failure-related death; only 50% of DCM patients survive more than 5 years beyond their initial diagnosis [1, 2]. Coronary artery disease, viral myocarditis, thyroid disease, immunologic processes, and toxins are known causes of DCM; however, the underlying pathology is not known in most cases [3–5]. In a population-based study, the prevalence of DCM was estimated to be 36.5 cases per 100,000, and 20–50% of these cases are familial [6–8]. Candidate gene analysis revealed that the cardiac actin encoding gene *ACTC1* mutations were the first sarcomeric gene mutations that caused DCM [9]. To date, mutations have been found in at least six genes encoding sarcomeric proteins: *β*-myosin heavy chain, cardiac myosin binding protein C, titin, cardiac actin, *α*-tropomyosin, cTnT, and cTnC [9–15]. 

The *TNNT2* gene (OMIM number *191045) encodes the protein cardiac TnT, which contains 15 exons and spans 25 kb on chromosome 1q32 [16]. Mutations in the *TNNT2* gene can cause three phenotypically distinct cardiomyopathies: hypertrophic, restrictive, and dilated [10, 17–19]. *TNNT2 *mutations are responsible for approximately 15% of all cases of familial hypertrophic cardiomyopathy (HCM) [20–22]. Recent data indicated that *TNNT2* mutations are also associated with DCM, and the overall frequency of *TNNT2* mutations in familial DCM is approximately 3–6% [23, 24].

Single nucleotide polymorphisms (SNPs) are the most common type of genetic variation in the human genome, and two recent large-scale SNP screens in European patients with DCM showed that SNPs in several genes were associated with DCM [25, 26]. Based on the above findings, we hypothesized that some cases of DCM are associated with specific polymorphisms in the *TNNT2* gene. To test this hypothesis and further understand the pathogenesis of DCM, we investigated 10 tagging SNPs in the *TNNT2* gene in DCM patients and normal control subjects from a Chinese Han population using matrix-assisted laser desorption/ionization time-of-flight mass spectrometry (MALDI-TOF-MS) techniques. Our results indicated that the SNPs rs3729547 and rs3729843 in the *TNNT2* gene were associated with DCM in the Chinese population, suggesting that the *TNNT2* polymorphisms may play an important role in susceptibility to DCM in the Chinese population.

## 2. Materials and Methods

### 2.1. Subjects and Selection of Tagging SNPs

This case-control study enrolled 97 unrelated DCM patients from the Fuwai Hospital. The clinical diagnosis was made in accordance with the revised criteria [1]. A total of 189 healthy unrelated individuals from a routine health survey were enrolled as controls. Patients with a history of hypertension, coronary heart disease, cardiac valve disease, diabetes, acute viral myocarditis, systemic diseases of putative autoimmune origin, and family history of DCM were intentionally excluded. This study was approved by the Ethics Committee of our hospital; the subjects involved were all of Han nation in the North of China and were informed of the study aims and provided written informed consent prior to participating.

 Genotype data on the *TNNT2* gene from the Han Chinese in Beijing (CHB) population were downloaded from the phase 2 HapMap SNP database (available at http://www.hapmap.org/), and tagging SNPs were selected in the Haploview software (available at http://www.broadinstitute.org/haploview) using a minor allele frequency (MAF) cutoff of 0.05 and a correlation coefficient (*r* ^2^) threshold of 0.8.

### 2.2. Isolation of DNA and Genotyping by MALDI-TOF-MS

Blood samples were collected from patients using tubes containing ethylenediaminetetraacetic acid. Genomic DNA was isolated from whole blood with a QIAamp DNA Blood Mini Kit (Qiagen, Germany). Genotyping was performed by MALDI-TOF-MS as described previously [27]. SNP genotyping was performed using a MassARRAY system (Sequenom, San Diego, CA, USA) based on the MALDI-TOF-MS method, according to the manufacturer's instructions. Completed genotyping reactions were spotted onto spectroCHIP (Sequenom) using a MassARRAY Nanodispenser (Sequenom), and the genotype was determined by MALDI-TOF-MS. Genotype calling was performed in real time with MassARRAY RT software version 3.1 (Sequenom) and analyzed using MassARRAY Typer software version 4.0 (Sequenom) (Table 1). 

### 2.3. Statistical Analyses

Differences in the distributions of selected variables and *TNNT2 *genotypes between the cases and controls were evaluated using the *χ* ^2^ test. The correlations between the *TNNT2 *genotype and the risk of DCM were estimated by computing the odds ratios (ORs) and the 95% confidence intervals (CIs) using logistic regression analysis. The *χ* ^2^ test was used to test for the Hardy-Weinberg equilibrium to compare the observed and expected genotype frequencies among the control subjects. All statistical analyses were performed with SPSS 13.0. All tests were two-tailed, and the significance was set at *P* < 0.05.

## 3. Results

The gender and age distributions of the DCM patients and the control subjects were compared with the Pearson's chi-square test and Student's *t*-test, respectively, and no significant differences were detected (control: *n* = 189, 54.0 ± 3.6 years, male/female = 150/39; DCM: *n* = 97, 51.6 ± 12.0 years, male/female  =  75/22, *P* > 0.05). 

The observed and expected genotype frequencies of each SNP were compared with the chi-squared test in DCM patients and the control subjects separately, and no significance was detected in either group. These results indicate that the samples fit the assumption of the Hardy-Weinberg equilibrium. The DNA variants and the Hardy-Weinberg equilibrium test of the 10 tagging SNPs in the DCM patients and control subjects were shown in Table 2.

**Figure 1:**
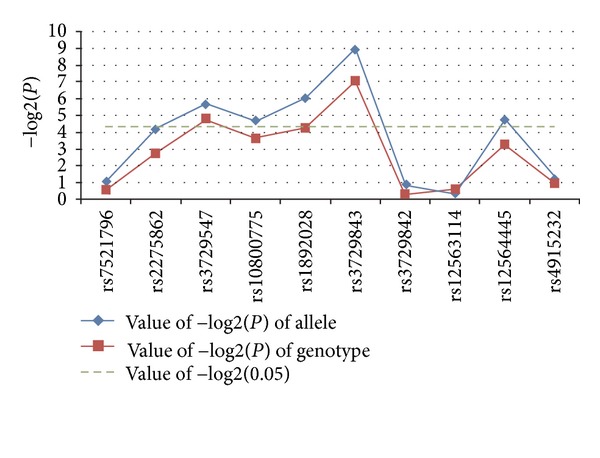
Mapping of the significance of each tagging SNP in the *TNNT2* gene. The *x*-axis shows the genomic position, and the *y*-axis shows the negative logarithm of the *P* value for each allele or genotype of each SNP.

Using the chi-squared test, we compared the genotype and allele frequencies in the *TNNT2* gene between the DCM patients and control subjects. Our results showed that the allele frequencies of the tagging SNPs rs3729547 (*χ* ^2^ = 5.474,  *P* = 0.019), rs1892028 (*χ* ^2^ = 5.855,   *P* = 0.016), rs3729843 (*χ* ^2^ = 9.620,  *P* = 0.002), rs12564445 (*χ* ^2^ = 4.351, *P* = 0.037), and rs10800775 (*χ* ^2^ = 4.252,  *P* = 0.039) were significantly correlated with DCM. However, among the genotypes, only those of the tagging SNPs rs3729547 (*χ* ^2^ = 6.63,   *P* = 0.036,  OR = 0.650, 95% CI = 0.453–0.934) and rs3729843 (*χ* ^2^ = 9.787,  *P* = 0.008, OR = 1.912, 95% CI = 1.265–2.890) had a significant correlation with DCM in the Chinese population. The allele and genotype frequencies of the ten tagging SNPs in the DCM patients and control subjects and the statistical analysis results were shown in Table 3 and Figure 1.

Because the great majority of DCM patients are male [1–5], we compared the frequencies of the genotypes of SNPs rs3729547, rs3729843, and rs10927875 in *TNNT2 *between the DCM patients and control subjects stratified by gender. In males, the distributions of the SNP rs3729547 genotypes were not significantly different between the DCM patients and control subjects, but the distributions of the SNP rs3729843 genotype were significantly different in the DCM patients and control subjects (*χ* ^2^ = 8.102, *P* = 0.017). In females, the distributions of the genotypes of rs3729843 and rs3729547 were not significantly different in the DCM patients and control subjects. 

**Figure 2:**
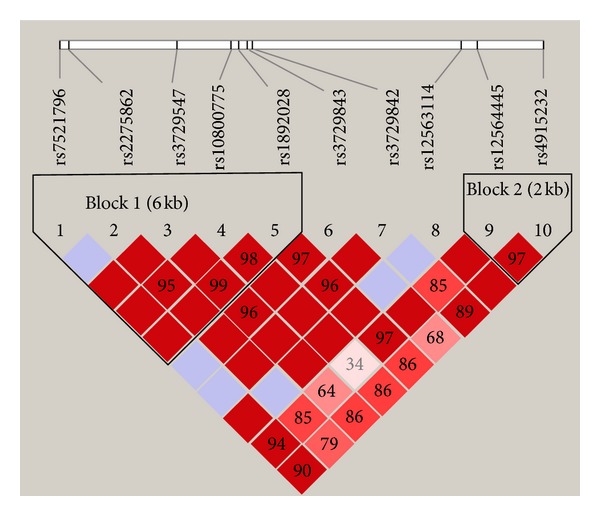
Pairwise linkage disequilibrium (LD) values calculated between tagging SNPs in the *TNNT2* gene. The value within each diamond represents the pairwise correlation between tagging SNPs (measured as *D*′), defined by the upper left and the upper right sides of the diamond. The diamond without a number corresponds to *D*′ = 1. Shading represents the magnitude and significance of the pairwise LD with darker red reflecting higher LD values and white indicating lower LD values.

The nonrandom associations between polymorphic variants at different loci on the *TNNT2* gene were then measured by the degree of linkage disequilibrium (LD). LD analysis showed that the SNPs rs7521796, rs2275862, rs3729547, rs10800775, and rs1892028 in the *TNNT2* gene, which are approximately 6 kb apart (block 3, Figure 2), were in high LD in the DCM patients (Figure 2, *D*′ > 0.80). The haplotype analysis showed that ACTCA (*χ* ^2^ = 6.66, *P* = 0.0099) and AGCTG (*χ* ^2^ = 4.003, *P* = 0.0454) in block 3 and AG (*χ* ^2^ = 3.988, *P* = 0.0458) in block 4 (rs12564445 and rs4915232) of the *TNNT*2 gene correlated significantly with DCM (Figure 2, Table 4).

## 4. Discussion

To our knowledge, this is the first study to show an association between DCM and SNPs in the *TNNT2* gene in the Chinese population. DCM is regarded as a heterogeneous disease. The present study shows that, in at least a subgroup of DCM patients, the SNPs in the *TNNT2* (rs3729547 and rs3729843) gene may be involved in the pathogenesis of DCM. 

DCM represents the third most frequent cause of heart failure and the most frequent cause of heart transplantation. Among patients with the so-called idiopathic DCM, 20–50% of cases are of genetic origin [6, 7]. Over the past decade, de novo mutations have been found in more than 30 genes encoding essential sarcomeric, cytoskeletal, and nuclear proteins in DCM patients [28], and mutations in the *TNNT2* gene have been found to be associated with familial HCM and DCM [10, 17–19, 23, 24]. 

Recent studies have suggested that cardiac TnT is essential not only for the structural integrity of the troponin complex but also for sarcomere assembly and cardiac contractility [22]. The troponin complex is a calcium sensor that regulates the contraction of striated muscle, and TnT is important in mediating the interaction between tropomyosin and actin and the rest of the troponin complex, which appears to modulate the activation of actomyosin ATPase activity and force [29]. Countless studies in reconstituted systems have provided valuable information on the functional effects of disease-associated mutations in TnT. The most extensively studied DCM-associated TnT mutation to date is ΔK210; functional studies of the ΔK210 mutation showed that the mutated protein reduced the Ca^2+^ sensitivity of actomyosin ATPase activity, which resulted in a decreased maximum speed of muscle contraction [30, 31]. Thus, DCM mutations in the troponin complex may induce a profound reduction in force generation, leading to impaired systolic function and cardiac dilation. 

In this study, we assessed whether polymorphism within the *TNNT2* gene might affect DCM susceptibility by comparing ten tagging SNP loci in DCM patients and normal control subjects. The representative SNP in a region of the genome with high linkage disequilibrium is called a tagging SNP. Among the ten tagging SNPs in the *TNNT2* gene, we found a significant association between the genotypes of rs3729547 (synonymous variant) and rs3729843 (noncoding SNP) and DCM. Although the allele frequencies of five tagging SNPs (rs3729547, rs3729843, rs1892028, rs1256445, and rs10800775) were significantly associated with DCM, the genotypes of rs1892028, rs1256445, and rs10800775 were not significantly associated with DCM, possibly because of the limited number of patients enrolled in the present study. LD analysis of the polymorphic SNPs observed in our study revealed a group of five SNPs, rs7521796, rs2275862, rs3729547, rs10800775, and rs1892028, located 6 kb apart; these alleles were in high LD and associated with DCM risk. As the majority of SNPs are likely to be allelic variants that do not affect expression or function of a protein, such SNPs are commonly used as genetic markers to localize nearby disease-causing variations in linkage and association analyses. SNPs that directly influence phenotype may be located within coding or regulatory regions of genes. SNPs within regulatory regions tend to have more quantitative effects, for example, by altering the expression level of a receptor or signaling protein, and result in a more subtle variation in the associated phenotype [32]. Recently, study showed that polymorphism in intron 3 of *TNNT2* significantly affected the mRNA expression pattern by skipping exon 4 during splicing in cardiomyopathy patients [33]. Missing exon 4 in cardiac troponin T is corresponding to isoforms cTnT2 and cTnT4, and the two isoforms increase might be related to hemodynamic stress [34]. These results in our study suggest that *TNNT2 *gene polymorphism, as like genetic markers to localize nearby disease-causing variations in linkage and association analyses, may play an important role in DCM susceptibility in the Chinese Han population. However, further functional analyses are needed to confirm the role of these polymorphisms in the pathogenesis of DCM.

In the present study, we have provided the evidence that shows that SNPs in the *TNNT2* gene may be implicated in the pathogenesis of DCM in a Chinese population. However, because the frequencies of genetic polymorphisms vary greatly among ethnic populations, further studies in other populations are needed to exclude a population-oriented association. In addition, the outcomes of the present study may be influenced by the limited sample size; larger studies are therefore required to investigate the potential associations between the SNPs in the* TNNT2* gene and the DCM susceptibility.

## Figures and Tables

**Table 1:** Sequences of the PCR primers used to genotype SNPs in the DCM patients and control subjects.

Markers	Forward primer (5′-3′)	Reverse primer (5′-3′)	Amplicon size (bp)	Temp. (°C)	GC (%)
rs7521796	TGCCAACAGAGAGGTGCTTC	CTTGAGGCTCAGCCTAATTG	93	46.5	56.3
rs2275862	AATATGAGGTGGGCCGCCAT	TATTACCGGACCCAGTGAAC	99	48.4	52.9
rs3729547	GAAGGACCTGAATGAGTTGC	AGAAACGAGCTCCTCCTCCT	99	50.2	60
rs10800775	AATCCCCTCCCAGGTCTTTG	TCATGTCATCAGCTTCTGCC	98	50.7	47.4
rs1892028	AGAGGGGACCATTGTCCAG	TCTAGGAGCTTCATGTGTGG	100	48	62.5
rs3729843	TCAAGGTCCTTGTTCTGAGC	TCTTGGCTAGGGCTTATCTG	99	47.1	44.4
rs3729842	TCAACGTTTGTTGATTGGGC	AGAACAGGCTTTCCCATGTG	99	46.7	31.8
rs12563114	TGGAAGGGCAGAGTAGGAGA	AATTCTCAGAGGAACCGTGC	100	45.2	44.4
rs12564445	AACTCGGAGACTGTTTCTAC	CTCTCTGACTTCAGTTAACC	95	47.7	47.1
rs4915232	CAATCTCGCTATTCTCTGCC	AGAAGAGTTTGAGGACTGGG	95	48.6	62.5

**Table 2:** Identified DNA variants and the Hardy-Weinberg equilibrium of 10 SNPs in the *TNNT2* gene in the DCM patients and control subjects.

Markers	Location of nucleotide change	Amino acid change	Note	Obs HET	Expt HET	HWE (*P*)	MAF
rs7521796	Intron 201330019 A>G	Noncoding	Novel noncoding SNP	0.115	0.115	1	0.061
rs2275862	Intron 201330366 C>G	Non-coding	Novel non-coding SNP	0.329	0.319	0.7683	0.199
rs3729547	201334382 T>C	Synonymous Ile [I]	Reported synonymous	0.504	0.484	1	0.41
rs10800775	Intron 201336386 C>T	Non-coding	Novel non-coding SNP	0.474	0.448	0.771	0.339
rs1892028	Intron 201336641 A>G	Non-coding	Novel non-coding SNP	0.489	0.498	0.7396	0.47
rs3729843	Intron 201336984 G>A	Non-coding	Reported non-coding SNP	0.285	0.331	0.5949	0.21
rs3729842	Intron 201337170 C>T	Non-coding	Reported non-coding SNP	0.225	0.241	0.3594	0.14
rs12563114	Intron 201344908 C>T	Non-coding	Novel non-coding SNP	0.081	0.084	0.5638	0.044
rs12564445	Intron 201345487 G>A	Non-coding	Novel non-coding SNP	0.435	0.427	0.7891	0.309
rs4915232	5′ near gene 201347946 A>G	Non-coding	Novel non-coding SNP	0.535	0.499	0.7407	0.482

Note: Obs HET: observed heterozygosity, Expt HET: expected heterozygosity, HWE (*P*): *P* value from the Hardy-Weinberg equilibrium test, and MAF: minor allele frequency.

**Table 3:** Genotype and allele frequencies of the SNPs from the *TNNT2* gene in the DCM patients and control subjects.

Marker	Genotype			*χ* ^2^, *P* value	Allele		*χ* ^2^, *P *value	OR (95% CI)
rs7521796	A/A	A/G	G/G		A	G		1.303 (0.613–2.772)
Patients	87 (0.897)	10 (0.103)	0 (0)	*χ* ^2^ = 0.747	184 (0.948)	10 (0.052)	*χ* ^2^ = 0.4751	
Controls	165 (0.873)	23 (0.122)	1 (0.005)	*P* = 0.688	353 (0.934)	25 (0.066)	*P* = 0.4906	
rs2275862	C/C	C/G	G/G		C	G		1.562 (0.987–2.471)
Patients	69 (0.711)	26 (0.268)	2 (0.021)	*χ* ^2^ = 3.802	164 (0.845)	30 (0.155)	*χ* ^2^ = 3.669	
Controls	113 (0.598)	68 (0.360)	8 (0.042)	*P* = 0.149	294 (0.778)	84 (0.222)	*P* = 0.055	
rs3729547	C/C	T/C	T/T		C	T		0.650 (0.453–0.934)
Patients	8 (0.084)	49 (0.516)	38 (0.4)	*χ* ^2^ = 6.63	65 (0.342)	125 (0.658)	*χ* ^2^ = 5.474	
Controls	37 (0.196)	94 (0.497)	58 (0.307)	**P** = 0.036	168 (0.444)	210 (0.556)	**P** = 0.019	
rs10800775	C/C	C/T	T/T		C	T		1.486 (1.019–2.169)
Patients	47 (0.49)	44 (0.458)	5 (0.052)	*χ* ^2^ = 5.024	138 (0.719)	54 (0.281)	*χ* ^2^ = 4.252	
Controls	74 (0.392)	91 (0.481)	24 (0.127)	*P* = 0.081	239 (0.632)	139 (0.368)	**P** = 0.039	
rs1892028	A/A	A/G	G/G		A	G		1.578 (1.090–2.291)
Patients	30 (0.357)	42 (0.5)	12 (0.143)	*χ* ^2^ = 5.947	102 (0.607)	66 (0.393)	*χ* ^2^ = 5.855	
Controls	46 (0.253)	88 (0.484)	48 (0.264)	*P* = 0.051	180 (0.495)	184 (0.505)	**P** = 0.016	
rs3729843	A/A	A/G	G/G		A	G		1.912 (1.265–2.890)
Patients	12 (0.126)	30 (0.316)	53 (0.558)	*χ* ^2^ = 9.787	54 (0.284)	136 (0.716)	*χ* ^2^ = 9.620	
Controls	7 (0.037)	51 (0.270)	131 (0.693)	**P** = 0.008	65 (0.172)	313 (0.828)	**P** = 0.002	
rs3729842	C/C	C/T	T/T		C	T		1.158 (0.697–1.925)
Patients	74 (0.763)	21 (0.216)	2 (0.021)	*χ* ^2^ = 0.381	169 (0.871)	25 (0.129)	*χ* ^2^ = 0.322	
Controls	139 (0.739)	43 (0.229)	6 (0.032)	*P* = 0.827	321 (0.854)	55 (0.146)	*P* = 0.571	
rs12563114	C/C	C/T	T/T		C	T		0.899 (0.390–2.07)
Patients	87 (0.906)	9 (0.094)	0 (0)	*χ* ^2^ = 0.828	183 (0.953)	9 (0.047)	*χ* ^2^ = 0.063	
Controls	174 (0.921)	14 (0.074)	1 (0.005)	*P* = 0.661	362 (0.958)	16 (0.042)	*P* = 0.8022	
rs12564445	A/A	A/G	G/G		A	G		0.663 (0.450–0.977)
Patients	6 (0.062)	37 (0.381)	54 (0.557)	*χ* ^2^ = 4.503	49 (0.253)	145 (0.747)	*χ* ^2^ = 4.351	
Controls	20 (0.106)	87 (0.463)	81 (0.431)	*P* = 0.105	127 (0.338)	249 (0.662)	**P** = 0.037	
rs4915232	A/A	A/G	G/G		A	G		1.155 (0.816–1.635)
Patients	25 (0.258)	55 (0.567)	17 (0.175)	*χ* ^2^ = 1.385	105 (0.541)	89 (0.459)	*χ* ^2^ = 0.6590	
Controls	46 (0.246)	97 (0.519)	44 (0.235)	*P* = 0.500	189 (0.505)	185 (0.495)	*P* = 0.4170	

**Table 4:** Haplotype analysis of SNPs in *TNNT2* gene between the DCM patients and control subjects.

	Haplotype	Frequency (DCM patients)	Frequency (control subjects)	*χ* ^2^	*P *value
Block 1	ACTCA	0.604	0.489	6.66	**0.0099**
	AGCTG	0.145	0.215	4.003	**0.0454**
	ACCTG	0.136	0.150	0.208	0.6485
	ACCCG	0.058	0.070	0.312	0.5762
	GCTCG	0.052	0.066	0.436	0.5089
Block 2	GA	0.541	0.498	0.937	0.333
	AG	0.252	0.333	3.988	**0.0458**
	GG	0.207	0.163	1.699	0.1925

## References

[1] Maron BJ, Towbin JA, Thiene G (2006). Contemporary definitions and classification of the cardiomyopathies: an American Heart Association Scientific Statement from the Council on Clinical Cardiology, Heart Failure and Transplantation Committee; Quality of Care and Outcomes Research and Functional Genomics and Translational Biology Interdisciplinary Working Groups; and Council on Epidemiology and Prevention. *Circulation*.

[2] Towbin JA, Bowles NE (2002). The failing heart. *Nature*.

[3] Franz WM, Müller OJ, Katus HA (2001). Cardiomyopathies: from genetics to the prospect of treatment. *Lancet*.

[4] Schönberger J, Seidman CE (2001). Many roads lead to a broken heart: the genetics of dilated cardiomyopathy. *American Journal of Human Genetics*.

[5] Seidman JG, Seidman C (2001). The genetic basis for cardiomyopathy: from mutation identification to mechanistic paradigms. *Cell*.

[6] Michels VV, Moll PP, Miller FA (1991). The frequency of familial dilated cardiomyopathy in a series of patients with idiopathic dilated cardiomyopathy. *New England Journal of Medicine*.

[7] Burkett EL, Hershberger RE (2005). Clinical and genetic issues in familial dilated cardiomyopathy. *Journal of the American College of Cardiology*.

[8] Mestroni L, Rocco C, Gregori D (1999). Familial dilated cardiomyopathy: evidence for genetic and phenotypic heterogeneity. *Journal of the American College of Cardiology*.

[9] Olson TM, Michels VV, Thibodeau SN, Tai YS, Keating MT (1998). Actin mutations in dilated cardiomyopathy, a heritable form of heart failure. *Science*.

[10] Kamisago M, Sharma SD, DePalma SR (2000). Mutations in sarcomere protein genes as a cause of dilated cardiomyopathy. *New England Journal of Medicine*.

[11] Shimizu M, Ino H, Yasuda T (2005). Gene mutations in adult Japanese patients with dilated cardiomyopathy. *Circulation Journal*.

[12] Gerull B, Gramlich M, Atherton J (2002). Mutations of TTN, encoding the giant muscle filament titin, cause familial dilated cardiomyopathy. *Nature Genetics*.

[13] Olson TM, Kishimoto NY, Whitby FG, Michels VV (2001). Mutations that alter the surface charge of alpha-tropomyosin are associated with dilated cardiomyopathy. *Journal of Molecular and Cellular Cardiology*.

[14] Li D, Czernuszewicz GZ, Gonzalez O (2001). Novel cardiac troponin T mutation as a cause of familial dilated cardiomyopathy. *Circulation*.

[15] Mogensen J, Murphy RT, Shaw T (2004). Severe disease expression of cardiac troponin C and T mutations in patients with idiopathic dilated cardiomyopathy. *Journal of the American College of Cardiology*.

[16] Gomes AV, Barnes JA, Harada K, Potter JD (2004). Role of troponin T in disease. *Molecular and Cellular Biochemistry*.

[17] Mogensen J, Kubo T, Duque M (2003). Idiopathic restrictive cardiomyopathy is part of the clinical expression of cardiac troponin I mutations. *Journal of Clinical Investigation*.

[18] Thierfelder L, Watkins H, MacRae C (1994). *α*-Tropomyosin and cardiac troponin T mutations cause familial hypertrophic cardiomyopathy: a disease of the sarcomere. *Cell*.

[19] Townsend PJ, Farza H, MacGeoch C (1994). Human cardiac troponin T: identification of fetal isoforms and assignment of the *TNNT2* locus to chromosome 1q. *Genomics*.

[20] Palm T, Graboski S, Hitchcock-DeGregori SE, Greenfield NJ (2001). Disease-causing mutations in cardiac troponin T: identification of a critical tropomyosin-binding region. *Biophysical Journal*.

[21] García-Castro M, Reguero JR, Batalla A (2003). Hypertrophic cardiomyopathy: low frequency of mutations in the *β*-myosin heavy chain (*MYH7*) and cardiac troponin T (*TNNT2*) genes among Spanish patients. *Clinical Chemistry*.

[22] Sehnert AJ, Huq A, Weinstein BM, Walker C, Fishman M, Stainier DYR (2002). Cardiac troponin T is essential in sarcomere assembly and cardiac contractility. *Nature Genetics*.

[23] Chang AN, Parvatiyar MS, Potter JD (2008). Troponin and cardiomyopathy. *Biochemical and Biophysical Research Communications*.

[24] Hershberger RE, Pinto JR, Parks SB (2009). Clinical and functional Characterization of *TNNT2* mutations identified in patients with dilated cardiomyopathy. *Circulation*.

[25] Villard E, Perret C, Gary F, Proust C, Dilanian G, Hengstenberg C (2011). A genome-wide association study identifies two loci associated with heart failure due to dilated cardiomyopathy. *European Heart Journal*.

[26] Stark K, Esslinger UB, Reinhard W (2010). Genetic association study identifies HSPB7 as a risk gene for idiopathic dilated cardiomyopathy. *PLoS Genetics*.

[27] Schaeffeler E, Zanger UM, Eichelbaum M, Asante-Poku S, Shin JG, Schwab M (2008). Highly multiplexed genotyping of thiopurine S-methyltransferase variants using MALDI-TOF mass spectrometry: reliable genotyping in different ethnic groups. *Clinical Chemistry*.

[28] Dellefave L, McNally EM (2010). The genetics of dilated cardiomyopathy. *Current Opinion in Cardiology*.

[29] Potter JD, Sheng Z, Pan BS, Zhao J (1995). A direct regulatory role for troponin T and a dual role for troponin C in the Ca^2+^ regulation of muscle contraction. *Journal of Biological Chemistry*.

[30] Morimoto S, Lu QW, Harada K (2002). Ca^2+^-desensitizing effect of a deletion mutation ΔK210 in cardiac troponin T that causes familial dilated cardiomyopathy. *Proceedings of the National Academy of Sciences of the United States of America*.

[31] Robinson P, Mirza M, Knott A (2002). Alterations in thin filament regulation induced by a human cardiac troponin T mutant that causes dilated cardiomyopathy are distinct from those induced by troponin T mutants that cause hypertrophic cardiomyopathy. *Journal of Biological Chemistry*.

[32] Ho E, Bhindi R, Ashley EA, Figtree GA (2011). Genetic analysis in cardiovascular disease: a clinical perspective. *Cardiology in Review*.

[33] Komamura K, Iwai N, Kokame K (2004). The role of a common *TNNT2* polymorphism in cardiac hypertrophy. *Journal of Human Genetics*.

[34] Mesnard-Rouiller L, Mercadier JJ, Butler-Browne G (1997). Troponin T mRNA and protein isoforms in the human left ventricle: pattern of expression in failing and control hearts. *Journal of Molecular and Cellular Cardiology*.

